# Universal antiretroviral therapy for HIV-infected children: a review of the benefits and risks to consider during implementation

**DOI:** 10.7448/IAS.20.1.21552

**Published:** 2017-06-27

**Authors:** Linda Barlow-Mosha, Victor Musiime, Mary-Ann Davies, Andrew J. Prendergast, Philippa Musoke, George Siberry, Martina Penazzato

**Affiliations:** ^a^ Clinic Department, Makerere University John Hopkins University (MUJHU) Research Collaboration, Kampala, Uganda​; ^b^ Department of Paediatrics and Child Health, Makerere University College of Health Sciences, Kampala, Uganda; ^c^ Research Department, Joint Clinical Research Centre, Kampala, Uganda​; ^d^ School of Public Health and Family Medicine, University of Cape Town, Cape Town, South Africa; ^e^ Blizard Institute, Queen Mary University of London, UK; ^f^ Research Department, Zvitambo Institute for Maternal and Child Health Research, Harare, Zimbabwe​; ^g^ Department of International Health, Johns Hopkins Bloomberg School of Public Health, Baltimore, MD, USA; ^h^ Maternal and Pediatric Infectious Disease Branch, National Institutes of Health, Bethesda, MD, USA; ^i^ HIV Department, World Health Organization, Geneva, Switzerland​

**Keywords:** Universal antiretroviral therapy, children, adolescents, resource limited settings, review, Sub Saharan Africa, WHO guidelines

## Abstract

**Background**: The 2016 World Health Organization (WHO) consolidated guidelines on the use of antiretroviral drugs for treating and preventing HIV infection, recommended to start all HIV-infected children on antiretroviral therapy (ART). Here, we explore the possible benefits and risks of implementing universal ART for all HIV-infected children and adolescents and outline some of the key considerations that led to the 2016 revision of WHO guidelines.

**Methods**: We conducted a review of the published data from 2000 to 2016, to ascertain the clinical and programmatic benefits, as well as the risks of implementing universal ART for all children.

**Results and discussion**: Universal ART for all children has the potential to increase treatment coverage, which in 2015 was only 51% globally, as well as providing several biological benefits, by preventing: premature death/loss to follow-up, progressive destruction of the immune system, poor growth and pubertal delay, poor neuro-cognitive outcomes and future burden to the health care system with complications of untreated HIV-infection. However, the strategy could be associated with risks, notably development of HIV drug resistance, antiretroviral drug toxicities and increased costs to an already stretched health system.

**Conclusion**: Overall, our findings suggest that the benefits could outweigh the risks and support universal ART for all HIV-infected children, but recognize that national programmes will need to put measures in place to minimize the risks if they choose to implement the strategy.

## Introduction

Survival of perinatally HIV-infected children has been transformed with the advent of antiretroviral therapy (ART). While access to ART has increased, only 51% of the 1.8 million children under 15 years living with HIV globally were receiving ART at the end of 2015, compared to 72% of HIV-infected pregnant women [1]. Whereas AIDS-related deaths have fallen for other age groups, mortality among HIV-infected adolescents rose by 50% from 2005 to 2012. In fact, AIDS is one of the leading causes of death among adolescents worldwide [2].

HIV infection progresses rapidly for the majority of children, with over 50% dying before two years of age in the absence of ART [3]. Little is known about the estimated one-fifth of children who may survive to up to 17 years without ART (so called “*the Slow progressors*”) [4], although it is clear that slow progression does not prevent HIV-related morbidity in the absence of treatment [5]. Most children living with HIV are infected either perinatally or in the first two years of life through breast-feeding, during a period of critical immune, neuro-cognitive and physical development. Without ART, HIV-infected children may experience growth and pubertal delay, long-term immune-dysfunction, neuro-developmental delay, and other long-term complications of chronic HIV infection [6].

The strategy to initiate ART in all HIV-infected children and adolescents irrespective of their clinical or immunological stage (universal ART), has been recommended in the 2016 World Health Organization (WHO) consolidated guidelines [7], and is expected to increase treatment coverage. We here review published data from the year 2000 to 2016, and discuss the potential benefits and risks that were considered during the WHO Guidelines development process leading to the most recent policy change.

## Methods

We searched for published journal articles of research conducted between the years 2000 and 2016. We included clinical trials and cohort studies for studies done to compare initiating ART among children immediately/shortly after diagnosis or deferring it until the children were at particular clinical or immunological thresholds. We also included cross-sectional and case control studies that compared or described outcomes between or among these two groups of children. Furthermore, we considered presentations at international conferences where the data had not been published at the time of the review.

We conducted a literature search in PubMed and embase electronic databases, as well as websites and reports of international HIV/AIDS conferences, and included the studies that fulfilled the search criteria. We particularly searched for relevant abstracts from the following conferences: International AIDS Conference; International AIDS Society conference on HIV pathogenesis and treatment; Conference on retroviruses and opportunistic infections (CROI); and International workshop on HIV Paediatrics. Studies were included if: they were conducted in the years of interest (2000 to 2016); and compared immediate or deferred initiation of antiretroviral therapy among children aged under 18years. There were no particular exclusions; specifically there were no exclusions with regard to language or geographical location. The search terms included: immediate or early antiretroviral therapy; deferred antiretroviral therapy; HIV infected infants and children; adverse events or toxicities of antiretroviral drugs; antiretroviral drug resistance. All references were imported into Endnote^TM^ (Clarivate analytics) and duplicates removed. This resulted in the 51 journal articles and 2 conference abstracts included in this review. The detailed search strategy is shown in the supplementary material (Table S1).

We also included some unpublished data at the time, from the leDEA cohort, after contacting the principal investigators accordingly.

The primary search was done by two of the authors (LBM and VM), who agreed on what was to be included; MP reviewed the selected articles and finalized and agreed with the two what was to be included. To this, the unpublished data from the leDEA cohort was added. All authors then reviewed the included studies and approved what is presented in this review.

## Results and discussion

The purpose of the review was to describe the benefits and risks of universal ART. Below is a description and discussion of our findings.

## Benefits of universal ART for children

### Post ART mortality

The survival benefit of immediate ART in infants has been clear since publication of the CHER trial: a randomized controlled trial (RCT) of HIV-infected infants at 6–12 weeks of age, which showed 75% mortality reduction in those starting immediate ART compared to deferring until immunologic or clinical progression to WHO 2006 ART initiation thresholds [8]. The only RCT in older children is the PREDICT study which recruited Thai children aged 1–12 years (median age 6.4 years) and showed no mortality benefit of immediate ART at CD4 15–24% versus deferring until a CDC Stage C event or CD4 < 15% [9]. However, the overall event rate was low so the study was ultimately underpowered to detect a difference in mortality or severe disease events, and there were few children in the younger age groups where the benefit of immediate ART is likely to be greatest.

**Figure 1.**
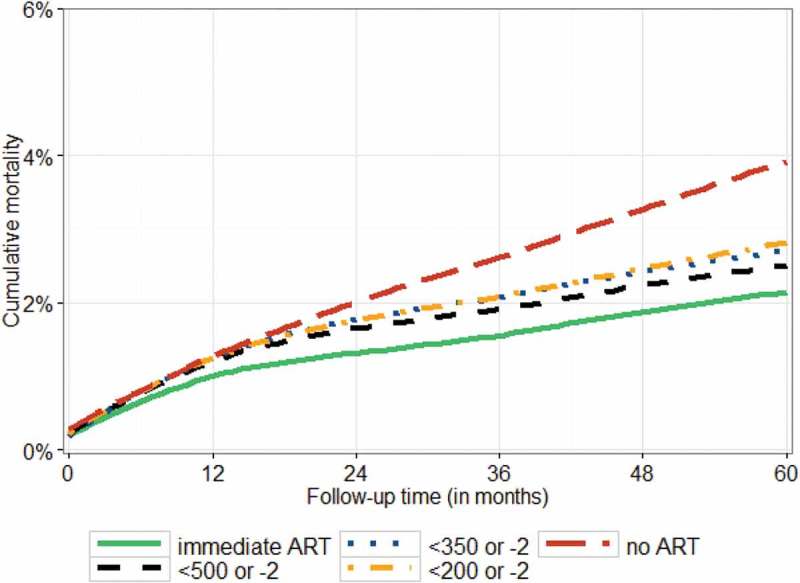
Mortality among children in Southern Africa, West Africa, and Europe with ART initiated at different CD4 thresholds: a causal modelling study [10].

Given the limited evidence on optimal timing of ART in children from RCTs, causal modelling is a valuable approach that uses observational data and simulates an RCT by adjusting for time-dependent confounding affected by prior treatment. A causal-modelling analysis from Southern Africa, West Africa and Europe showed a clear mortality benefit of immediate ART compared to deferring until immunological(CD4 < 500 cells/µl) or clinical (weight-for-age *z*-score (WAZ) <–2 in 5–10 year old children with initial CD4 > 500 cells/µl) progression, with a mortality difference of 0.4% (95% Confidence IntervaI [CI]: 0.02–0.6%) by 5 years of follow-up, as shown in Figure 1 [10]. There was no significant mortality benefit of immediate ART compared to deferring until a higher threshold of CD4 < 750 cells/µl or <25% in younger children (aged 1–5 years). Further, a previous analysis from Southern Africa showed that only 11% of children aged 1–5 years presented with CD4 > 750 cells/µl or CD4% > 25% and there was a 75% probability of CD4 decline to below this threshold in the next 3 years, supporting earlier ART initiation in this group [11].

Evidence regarding the mortality effects of earlier ART initiation in adolescents is even more limited as the PREDICT trial excluded those aged >12 years. The reduced morbidity with immediate versus deferred ART in adults in the START [12] and TEMPRANO [13] trials cannot be assumed to extend to adolescents due to differences in infection routes, lifestyle factors, adherence and puberty/growth effects. The causal-modelling analysis from Southern Africa, West Africa and Europe showed no mortality benefit of immediate compared to deferred (CD4 < 500 cells/µl) among 10–15year old adolescents; however, only 14% of adolescents in the 10-15 year age group presented with CD4 > 500cells/µl, and the population was a mix of both vertically and horizontally infected children with likely different effects of immediate ART. Importantly, all causal modelling analyses to date have consistently shown a clear trend towards lower mortality with earlier ART initiation, even though the mortality difference between immediate and deferred ART was not always statistically significant [10,11].

While mortality benefit would be the most important argument favouring universal ART, there are also important operational and programmatic considerations. In a systematic review, a significant difference was observed in characteristics of children initiating ART in developing compared to developed countries. Baseline CD4% were lower and viral loads higher in ART-naive children in developing countries when compared to those in developed countries (mean 12% and 5.5 log_10_ copies/ml compared to 23% and 4.7 log_10_ copies/ml, respectively) and mortality was higher (7.8% versus 1.6%) [14]. Low baseline CD4 count, low WAZ, and high viral loads (VL) were identified by individual studies as strong predictors of mortality in both settings. Mortality-rate differences between developing and developed countries persisted even after adjusting for baseline CD4 count, and were attributable to differences in WAZ and/or VL. In developing countries, children were older at the time of diagnosis and treatment initiation and had more advanced disease than those in developed countries; the majority of deaths occurred in the first 6 months of treatment [14]. Universal ART allows for all children to be started on ART as soon as they are identified as infected, before the onset of advanced disease with better outcomes likely, including reduced mortality, post ART initiation.

### Improved growth

There is a high frequency of both stunting (low height-for-age) and wasting (low weight-for-height) among HIV-infected children [15–17]. The mechanisms underlying growth failure are complex and multi-factorial; contributing factors include: inadequate calories, gastrointestinal and other opportunistic infections, HIV enteropathy, chronic inflammation, abnormal resting energy expenditure and endocrine abnormalities [17,18]. In HIV-infected children, the rate of growth, quantity of fat-free mass (FFM) and energy intake are closely related to the level of HIV replication [18]; therefore, the energy intake for HIV-infected children not on ART may be insufficient to support normal development of FFM and growth.

The PREDICT trial showed significantly faster height gain and greater mean height-for-age *z*-score (HAZ) with immediate compared to deferred ART, with a mean difference in HAZ of 0.22 by 144 weeks of follow-up [9]. Similarly causal modelling studies in children age 1–10 years show consistently better height gain with immediate compared to deferred ART [10,11]. For example in children aged 5–10 years with initial CD4 > 500cells/µl, immediate ART was associated with a HAZ difference of 0.1(95% CI: 0.07–0.12) by 4 years of follow-up compared to deferring until CD4 < 500 cells/µl [10]. While causal modelling has not shown significantly better height gain with immediate versus deferred ART in adolescents 10–16 years old, there is clearly a trend towards better growth with earlier ART [10]. However, it is important to note that height gains, even with earlier ART initiation, are only modest, and there is a high frequency of persistent stunting in HIV-infected children and adolescents.

In cohort studies improved growth on ART has also been seen; however, these effects vary by age at ART initiation. Better growth has been reported in children who initiated ART at younger compared to older ages [17,19], with children under 3 years being 2–3 times more likely to attain population norms for WAZ when compared to older children at ART initiation [19].

Similarly in the ARROW trial in Uganda and Zimbabwe, starting ART at older ages was associated with greater impairments in pre-ART HAZ in both males and females and pre-ART BMI-for-age in girls [20]. Similar findings have been reported in the IeDEA West African cohort, where adjusted catch-up growth was more likely for children <5 years of age at ART initiation compared to children ≥5 years [21].

### Pubertal development

HIV can affect growth and development of infants and children in many ways, including: the associated malnutrition, recurrent infections, and immune activation, all of which could affect the production of hormones that control growth and development. The ARROW trial highlights the importance of considering factors such as pubertal development, when making decisions about timing of ART initiation in older children [20]. In this study, delaying ART initiation until older childhood substantially delayed puberty and menarche, independently of immuno-suppression. Similar findings have been reported in the United States, where HIV-infected children (compared with HIV-exposed but uninfected children) had delayed onset of puberty, which was reduced with earlier initiation of ART [22].

### Immune recovery

A comparison of Ugandan and UK/Irish cohorts reported that younger children in both cohorts had better immunological, weight, and growth responses than older children at ART initiation [23]. In a study in Thailand, only 51% of children achieved immune recovery by 96weeks after ART initiation [24]; predictors of CD4 recovery were younger age, female sex, higher baseline CD4%, and sustained virologic suppression after week 24. In the ARROW trial, using models to predict long-term CD4 recovery, younger children (below 6 years of age) were more likely to achieve high CD4 counts later in adulthood compared to those ≥6 years. In addition, initiating ART in children >5 years based on the WHO criteria at the time would result in lower CD4 counts during adulthood compared to initiation under 5years. HIV-infected children remaining ART-naïve beyond 10 years would be unlikely to normalize their CD4 count [25]. A recent analysis of 4808 HIV-infected children followed up in the IeDEA cohort found that immune recovery was significantly lower in children initiating ART above 5 years of age [26]. Collectively, these findings suggest that initiating ART at younger ages, rather than waiting for clinical or immunological disease progression, leads to better long-term CD4 recovery, which is an important consideration for children given that they require lifelong treatment.

### Viral suppression and immune activation

Viral reservoirs that develop early in the infection prevent sterilizing immunity and are an obstacle to finding an HIV cure. Having cellular and anatomical reservoirs contributes to long-term persistence of HIV-1 [27]; with HIV-1 DNA integrate in cells in the central nervous system, male urogenital tract, and resting memory CD4+ T-cells. The half-life of this latent reservoir could last as long as 44 months and its eradication would require over 60 years of antiretroviral therapy [27]. Thus, these latently infected resting CD4+ T-cells provide a mechanism for life-long persistence of HIV-1 forms that are capable of replicating, making hopes of viral eradication with current antiretroviral regimens unattainable [27]. However, ART initiation before 6 months of age can potentially result in very low levels of markers of HIV persistence and minimal HIV-specific immune responses [28]. However, little is known about the extent of HIV reservoirs in perinatally HIV infected children initiated on ART in late childhood or adolescence.

### Neurodevelopment

A systematic review reporting neuro-developmental outcomes showed that HIV infection was associated with lower cognitive scores, but that ART appeared to eliminate most of these deficits [29]. Importantly, delays in ART initiation appeared to worsen cognitive impairments. Similar findings have been reported recently in Uganda where among HIV-positive children aged 0–6 years, longer duration on ART was associated with a reduced risk of impairment in fine motor, receptive language, expressive language and overall global early learning composite scores [30]. In another systematic review of neurodevelopment in older children and adolescents, HIV was associated with neuro-developmental delays; if ART was postponed improvements in neuro-developmental scores were less likely [31]. Although in the PREDICT trial, no neuro-cognitive benefits were observed for early versus deferred ART, HIV-infected children performed worse than HIV uninfected controls in all neurodevelopment tests performed [24], highlighting the long-term effects of HIV on neurodevelopment.

### Reduction in incidence of tuberculosis

HIV/TB co-infected children and adolescents are at increased risk of death and chronic lung sequelae, compared to those infected with HIV only, and interventions to reduce TB among HIV-infected children are urgently needed. ART may be of benefit to reduce the incidence of TB among children. A study in South Africa found a 63% decrease in incidence of culture-confirmed TB coinciding with an increase in ART coverage from 43% to 84% from 2005 to 2009 [32]. There was also an observed significant decline in TB hospitalizations among HIV-infected children. The reduction may in part have been due to reduced *Mycobacterium tuberculosis* transmission due to increased ART access and resultant decreased TB incidence among HIV-infected adults.

### Reduction in HIV-related long-term complications

Cardiac disease has been described in HIV-infected ART naive children, and the risk increases with age [33–35]. A multi-centre cohort study (PHACS) reported that long-term ART appeared cardioprotective for HIV-infected children and adolescents where those on ART had lower rates of cardiomyopathy than those without ART [36].

As children get older, their risk of developing HIV-associated chronic lung disease increases. Prior to access to ART, lymphocytic interstitial pneumonitis (LIP) was the predominant cause [37,38]. LIP responds well to ART, but delayed initiation of therapy can lead to long-term sequelae in adolescence [38]. I Initiation of ART during early childhood might be preventive of the disease, in addition to geographical location and genetic predisposition which could also play a role in its occurrence [39]. On the other hand, if ART is delayed until late childhood or adolescence, lung function is unlikely to improve [40,41].

### Programmatic benefits

Perhaps the most important benefit of universal ART in HIV-infected children and adolescents is the potential to improve treatment coverage. The WHO 2013 consolidated ART guidelines recommended disease staging and CD4 testing for HIV infected children above 5 years of age to determine ART eligibility [​^42^]. However, access to CD4 testing is still limited in most priority countries [43], and hence this requirement leads to delays in ART initiation. Furthermore, WHO disease staging is complex and may not always be feasible at lower level health facilities. Universal ART for all children and adolescents means removal of the CD4/WHO staging barrier, simplification of guidelines and ease of ART initiation in resource limited health facilities. While considering the WHO 2013 consolidated ART guidelines to provide universal ART for all HIV-infected children under 5 years, the Ministry of Health in Uganda, went further to recommend universal ART for all under 15 years in the national guidelines [44]. As a result, paediatric ART coverage increased from 22% in 2013 to 32% in 2014 [45]. Universal ART may also have the potential to improve retention in care, as suggested by data from Mozambique and Uganda, where children on ART were observed to be more likely to remain in care than the ART naive children [46,47]. Finally, harmonization with adults recommendations, based on the START and TEMPRANO trials [12,13], remain of great value for treatment programmes.

However, it is important to recognize that ART initiation is preceded by identification of the HIV-infected children. Therefore significant gains in ART coverage will only be possible if there is community mobilization and investment in HIV testing including early infant diagnosis.

## Risks of universal ART for children

### Antiretroviral drug toxicities

There is no doubt that ART is beneficial, but it is also associated with toxicities. Preclinical and clinical studies have demonstrated short-and long-term adverse events on ART, including haematological, renal, cardiovascular, bone and metabolic abnormalities [48,49]. The short term side effects are frequently observed on initiation of ART, with dizziness and gastrointestinal disorders (diarrhoea, nausea and vomiting) more commonly observed [50]. Dizziness and other central nervous system disorders (concentration problems, sleep disorder, psychotic reactions and seizures) are particularly observed with efavirenz [51], and potentially result in sub optimal ART adherence and subsequent ART failure [51,52]. Also the sleep disorders and poor concentration, observed with efavirenz, may affect learning/school performance, which would be undesirable effects in an otherwise well child. The long term effects are particularly important as they impact quality of life in adulthood. Lipodystrophy, as described by abnormalities of fat loss (lipoatrophy), fat accumulation (lipohypertrophy), dyslipidemia, insulin resistance, diabetes, lactic acidosis or mixed forms, has been observed to occur in 20–50% of patients on ART for prolonged periods. These abnormalities have been associated with specific antiretroviral drugs, such as stavudine, lopinavir/ritonavir, zidovudine and efavirenz; older age; puberty; and longer ART duration [20,53–57]. Furthermore, the nucleoside reverse transcriptase inhibitors (NRTI), lamivudine, abacavir, zidovudine, emtricitabine, and tenofovir were observed *in vitro* to significantly inhibit telomerase activity, with tenofovir being the most important inhibitor, with resultant shortening of the telomere length, a key indicator of ageing. In fact, peripheral blood mononuclear cells from HIV-infected patients receiving NRTI-containing ART had significantly lower telomerase activity than HIV-uninfected patients and HIV-infected patients receiving non-NRTI–containing ART. Telomere length was significantly inversely associated with age and importantly with the total duration on any NRTI [49]. It is, therefore, possible that use of NRTIs, which form the backbone of first and second line ART regimens, could cause early ageing in HIV-infected children. In advanced HIV disease, the clinical benefits of ART outweigh the toxic effects of antiretroviral drugs, but in children who are asymptomatic and especially with good immunological function, such as long term non progressors [58], early exposure to ART may have a different risk and benefit balance.

### Antiretroviral drug resistance

HIV-infected children >3 years of age initiating ART currently are recommended to start a combination of NRTIs and non nucleoside reverse transcriptase inhibitors (NNRTIs). The recommended NRTIs include abacavir, zidovudine, lamivudine and tenofovir, while the recommended NNRTIs are efavirenz and nevirapine [7]. These drugs have a low resistance barrier [59]. Indeed in children failing first-line ART in Africa, several resistance associated mutations, including multiple thymidine analogue mutations and dual class resistance, have been observed [60–62]. In Europe where ART has been used for a longer time among children, triple class failure was observed in 10% of 1007 children in a multi-cohort analysis, with higher risk associated with duration on ART and older age at ART initiation; notably only 24% of the children were triple class exposed [63]. As children stay on ART for longer, triple class resistance is a likely phenomenon in Africa and similar settings. Antiretroviral drug resistance could be exacerbated with universal ART due to the lack of motivation to take medications that could lead to poorer adherence in otherwise well children, especially among adolescents [64]. Furthermore, rapid introduction of universal ART without the necessary planning and preparation may increase the burden on busy health facilities and lead to lower quality of the services and potential drug stock outs. This may overall increase the risk of selecting drug resistance that would further limit future treatment options for the adolescents and babies that may be born to them [65].

### Implementation challenges and resulting risks

Increased treatment coverage is very important for survival among HIV-infected children. However, it is likely to exacerbate existing challenges for ART programmes in resource limited settings. Many HIV-infected children live in rural areas [66], where a reliable supply of HIV commodities and provision of psychosocial support in health facilities may be more difficult. In addition, infants may be relatively neglected due to the challenges of prioritizing Early Infant Diagnosis (EID) and early infant ART, which are considerably more complex than diagnosing and initiating ART in an older child [8]

As experienced in Uganda, universal ART increases the demand on supply chain systems and leads to more work for the limited human resources. Strengthening laboratory systems will be urgently needed, particularly for virological monitoring which is a critical tool to assess treatment response without baseline CD4 testing and for which access is still limited [43]. Overall, it is clear that the demand for HIV commodities, human resources and infrastructure will increase and so will the need for sustainable and reliable funding.

## Conclusions

While the evidence underscoring universal treatment for all children and adolescents is not as robust as in adults, an increasing body of evidence points towards reduction in mortality and morbidity, improvement of growth, prevention of neurodevelopmental and pubertal delays as well as reduction in end organ effects. These benefits don’t come without risks, however, and the attention of policy makers to ensure access to high quality HIV care will be needed to address these risks.

As the global community strives to achieve global treatment targets by 2020 and aspires to reach the fast-track targets for children and adolescents, more efforts will need to be in place to ensure that the quality of HIV services is improved and sustained. Measures are needed to enable timely and reliable viral load monitoring for early identification of virological failure; to ensure that infants at the highest risk of mortality are identified early and treated in a timely manner; to ensure reliable provision of child-friendly formulations and provide the most effective and tolerable regimens; to offer adequate psychosocial support and community based interventions to promote adherence; to ensure that adolescents’ health standards are applied and adolescents receive services and care that meet their needs.

Training and supportive supervision of health workers will be critical to this acceleration. This will not only empower them to manage paediatric and adolescent treatment, but also recognize and manage ART toxicity and ART failure while seeking advice via appropriate referral or consultation to higher levels of care when needed.
